# An Examination of Task‐Evoked fMRI Data Processing in Functional Connectivity

**DOI:** 10.1002/jnr.70132

**Published:** 2026-06-09

**Authors:** Alice Giubergia, Sara Mascheretti, Valentina Lampis, Tommaso Ciceri, Martina Villa, Chiara Andreola, Filippo Arrigoni, Alessandra Bertoldo, Denis Peruzzo

**Affiliations:** ^1^ Neuroimaging Unit Scientific Institute IRCCS Eugenio Medea Bosisio Parini Italy; ^2^ Department of Information Engineering University of Padova Padova Italy; ^3^ Department of Brain and Behavioral Sciences University of Pavia Pavia Italy; ^4^ Child Psychopathology Unit Scientific Institute IRCCS Eugenio Medea Bosisio Parini Italy; ^5^ Department of Psychological Sciences University of Connecticut Storrs Connecticut USA; ^6^ Institute for the Brain and Cognitive Sciences (IBACS) University of Connecticut Storrs Connecticut USA; ^7^ Haskins Laboratories New Haven Connecticut USA; ^8^ Université Paris Cité, Laboratoire de Psychologie de Développement et de L'éducation de L'enfant Paris France; ^9^ Buzzi Children's Hospital Milan Italy

**Keywords:** functional connectivity pipelines, machine learning, pseudo‐resting state, task‐fMRI

## Abstract

Although functional connectomics typically relies on resting‐state fMRI, its analytical methods have been applied to task fMRI data in the investigation of broader involvements of brain regions even if inactive during a specific task. The purpose of this study is to assess the feasibility of inferring a true resting‐state connectivity from task‐fMRI data and to investigate the impact of connectomic‐based analysis on behavioral trait studies. To this purpose, subjects underwent two visual fMRI tasks. The Blood‐Oxygen‐Level‐Dependent (BOLD) time‐series were processed to get both a “task” condition and a “pseudo‐resting” condition applying different task regression setups to derive connectomes. Stimulus‐classification experiments were conducted to compare “task” and “pseudo‐resting” connectomes. Additionally, the influence of task regression was assessed through a classification experiment comparing children with Developmental Dyslexia (DD) and Typical Readers (TR). While task regression successfully removes task‐related content from fMRI signals, stimulus information could still be inferred from connectomes, regardless of the preprocessing method used. Furthermore, a Support Vector Machine (SVM) experiment effectively discriminates between DD and TR in both “task” and “pseudo‐resting” conditions. The study explored the impact of preprocessing in task fMRI experiments analyzed with connectomics. The ability to classify the stimuli in “pseudo‐resting” conditions suggests that connectomes retain task‐related signals even after task regression. Discriminative connections vary across tasks, affecting how classifiers differentiate between DD and TR. Despite these task‐related differences, preprocessing had no effect on the inference of classification rules, indicating that key features are similarly evaluated in both tasks.

## Significance Statement

This study explores the contribution of task information to connectomics towards a methodological consensus for preprocessing among task‐state connectivity studies. The evaluation of resting‐state‐like connectivity inferred from task‐fMRI data revealed that connectomes retain task‐related information even after regressing task‐related content. However, connectomes from task‐fMRI can be used to investigate clinical conditions and their impact on brain connectivity. The findings suggest that brain connectivity patterns, critical for understanding cognitive functions, can be extracted from task data and applied to diverse populations. This has important implications for studying neurodevelopmental disorders, allowing for better insights into disease‐specific brain network alterations.

## Graphical abstract

In this work, we analyzed the preprocessing of task‐fMRI data to explore the contribution of the task information in connectomics towards a methodological consensus for preprocessing among task‐state connectivity studies. We investigated whether a task‐free connectivity can be inferred from task‐fMRI and examined its impact upon a clinical disorder.

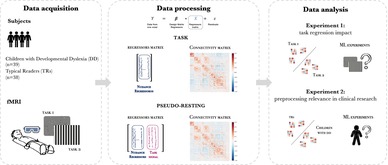

**Abbreviations**
BOLD: blood oxygen level dependent
CM: coherent motion
CV: cross validation
DD: developmental dyslexia
FC: functional connectivity
GLM: general linear model
HRF: hemodynamic response function
ICA: independent component analysis
PCA: principal component analysis
ROI: region of interest
SG: sinusoidal gratings
SVM: support vector machine
TR: typical reader

## 1 Introduction

Functional magnetic resonance imaging (fMRI) has emerged as a powerful tool for examining human brain connectivity underlying cognitive functioning and as an indirect and non‐invasive measurement of brain activity. Consequently, functional connectomics has gained significant attention in the neuroscientific community for investigating cognitive behaviors and their underpinning neural interactions.

An established method to evaluate spontaneous neural interactions involves estimating Functional Connectivity (FC), which assesses and analyses the statistical associations between brain regions based on their neural activity patterns (Friston 2011). Approaches for computing functional connectivity include seed‐based methods, where a reference voxel/Region Of Interest (ROI) is tested for functional correlation with the remaining voxels/ROIs (Chen and Glover 2015), and the data‐driven approaches, such as Independent Component Analysis (ICA) and Principal Component Analysis (PCA), which decompose the 4D fMRI data into latent factors, describing their temporal and spatial characteristics to identify and separate distinct functional brain networks (Chen and Glover 2015). Nevertheless, the most established way to perform FC is to measure the pairwise correlations among ROIs due to the spontaneous neuronal firing and unconstrained mental activity (Birn et al. 2013). This provides insights into the extent to which these regions are functionally connected in the brain (Friston 2011). The reliability and validity of FC measurements depend on standard preprocessing steps, which are crucial to improve data quality, reducing confounding factors that may distort the results, and ensuring that the signal time variability reflects the neural activity (Graff et al. 2022; Jiang et al. 2020). Conventionally, resting‐state based fMRI preprocessing includes correction of slice timings, magnetic inhomogeneity‐induced distortions and motion, regression of white matter (WM) and cerebro‐spinal fluid (CSF) signals, and spatial and temporal filtering (Chen and Glover 2015). Despite these steps, signal fluctuations from non‐neural activity (e.g., cardiac pulsation, head movements) can overestimate the strength of FC (Birn 2012; Power et al. 2012). Therefore, an important practical consideration is the amount of data, and consequently the acquisition time, required to obtain reproducible quantitative FC. The optimal scan duration to accurately differentiate the functional connectivity of an individual from a group of subjects using automated machine learning classifiers for resting‐state fMRI is between 12 and 20 min (Anderson et al. 2011; Wahab et al. 2022).

While FC was originally intended to portray resting‐state brain organization, recent developments in neuroimaging research extended the range of the FC approaches to explore task‐based fMRI data (Di and Biswal 2019). However, there is still a lack of consensus on the processing steps in this context. For example, several studies do not collect resting‐state data and seek to infer a “pseudo‐resting” state from task‐modulated data (Bhandari et al. 2020; Di and Biswal 2019). Much of the variance observed in task‐based fMRI experiments can be explained by the underlying resting‐state spontaneous neural activity (Arfanakis et al. 2000; Fair et al. 2007; Fox et al. 2006); however, no standard approach exists to derive the resting “substrate” of BOLD responses. Some studies remove task information by regression of task predictors (Fair et al. 2007), other studies use task‐modulated data studying those regions that are not affected by task‐related cognitive activities (Arfanakis et al. 2000) or map the well‐established resting networks (e.g., the Default Mode Network—DMN) to regressed task‐based data (Bhandari et al. 2020; Harris et al. 2014). When dealing with FC analysis of task‐based fMRI data, researchers tend to regress‐out task design with the assumption to remove any unique task‐specific variance from the time series (Varangis et al. 2021).

A recent work by Di and Biswal (2019) shows that task‐related brain activations amplify the trait‐relevant individual differences in functional connectivity patterns. Therefore, the removal of task‐related content from the fMRI signal in the computation of FC may highlight any interactions between the psychological context amplified by the task and the underlying neural process (Cole et al. 2019). However, there has been little discussion on how to process task‐evoked functional data to inspect differences in brain functional organization of human behaviors and cognition.

In this paper, we investigate the applicability of the FC approaches to task‐based fMRI data with two main objectives: (i) to assess whether a connectivity analysis can infer a “pseudo‐resting” condition, i.e., a connectome unrelated to the original task and, possibly, equivalent to one properly derived from resting‐state fMRI data; and (ii) to explore the impact of connectivity‐oriented data processing on subsequent analyses of differences in behavioral traits.

## 2 Materials and Methods

### 2.1 Participants

Seventy‐seven subjects (age range: 9–18 years, M/F: 49/28, 39 Typical Readers—TR and 38 with Developmental Dyslexia—DD) were recruited in a project aiming to study the genetic basis of DD (Riva et al. 2019; Mascheretti et al. 2024). Both TRs and children with DD underwent a neurophysiological assessment. The two groups showed significant differences in age, sex distribution, intelligence quotient (IQ), and ADHD‐related inattentive traits. Table 1 reports the descriptive statistics of demographic and neurological variables of both children with DD and TRs. The study was reviewed and approved by the Ethics Committee of the Scientific Institute, IRCCS Eugenio Medea.

**TABLE 1:** Descriptive statistics and neuropsychological variables.

	Children with DD (*n* = 39)	Typical readers (*n* = 38)	*χ* ^2^	df	*p*
Gender (female/male)	19/20	9/29	4.19	1	0.041
	**Mean (SD)**	**Mean (SD)**	***t*‐test**	**df**	***p***
Age	12.69 (1.64)	14.29 (1.67)	−4.23	75	< 0.0001
IQ^a^	13.18 (3.28)	11.82 (2.25)	2.12	75	0.037
ADHD‐Inattention^b^	48.21 (7.6)	60.47 (11.4)	−5.57	75	< 0.0001

^a^ As estimated by the Block design subtests of the WISC‐II (Cornoldi 1995).

^b^ As estimated by the DSM‐IV‐Inattention (DSM‐IV‐I) subscale of the CPRS‐RL (Conners et al. 1998; Conners et al. 2007; Cunningham and Stanovich 1997).

### 2.2 Experimental Procedure and fMRI Task Design

MRI data were collected on a 3 T Philips Achieva dStream scanner with a 32‐channel head coil. Visual stimuli were delivered through a VisuaStim digital device for fMRI (Resonance Technology Inc., Northridge, CA, USA). The MRI protocol included the acquisition of an anatomical T1‐weighted (T1W) 3D Turbo Field Echo sequence (TE: shortest (∼3.7 ms); TR: shortest (∼8.1 ms); voxel size: 1 × 1 × 1 mm^3^; FOV 256 × 256 × 175 mm^3^; FA: 8°) and two task fMRI sequences acquired with a T2*‐weighted Gradient Echo planar sequence (TE: 26 ms, TR: 2 s, voxel size: 3 × 3 × 3 mm^3^, FOV: 240 × 240 mm^2^; slice thickness: 3 mm; slice gap: 0.5 mm; slice number: 39; FA: 90°). The task‐fMRI sequences were acquired during the administration of two psychophysiological visual tasks, i.e., Sinusoidal Gratings (SG) and Coherent Motion (CM), with the aim of eliciting the dorsal stream pathway (both SG and CM) and the attention network (CM) (Mascheretti et al. 2021). The task‐fMRI sequence duration was 11′30″ and 9′30″ for the SG and the CM, respectively. The SG consisted of a full‐field sinusoidal grating with sinusoidal counter phase flicker, designed to elicit the dorsal stream pathway by simultaneously manipulating spatial and temporal frequencies and luminance contrast. The task included three different combinations of color, spatial frequency, and temporal frequency, each presented in blocks of 14 s. The CM was designed to assess the sensitivity to radial motion (expansion or contraction) coherence by presenting a fast stimulus (250 ms duration time) showing a gray background, populated with small white and black dots drifting at different coherence levels. The subject was asked to identify the direction of the coherent dots motion. For a detailed description of the tasks, please refer to Mascheretti et al. (2021).

### 2.3 MRI Data Common Preprocessing

**FIGURE 1:**
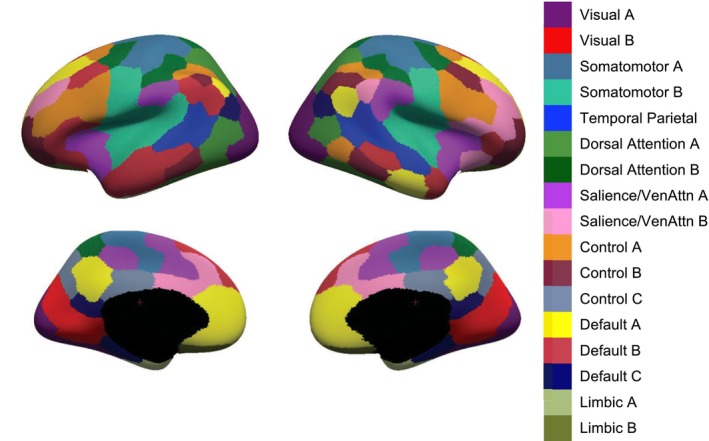
Functional parcellation. Yan 2023 200‐area functional parcellation matched to Yeo 2011 17 networks.

T1W images underwent the standard *recon‐all* pipeline of FreeSurfer (https://surfer.nmr.mgh.harvard.edu/) for cortical reconstruction and volumetric segmentation. Briefly, the processing stream includes skull stripping, transformation to Talairach space, intensity normalization, segmentation of the volumetric structures, and estimation of the white matter (WM)–gray matter (GM) interface (white surface) and of the GM–cerebrospinal fluid (CSF) interface (pial surface). The surfaces were parcellated according to the homotopic Yan 2023 atlas (Yan et al. 2023) to divide each hemisphere into 100 cortical regions, which can be grouped into 17 macro‐regions matched to the Yeo 2011 17 networks (Figure 1). Moreover, we took into account 18 regions of the deep gray matter (dGM) derived from the volumetric segmentation (Fischl et al. 2002, 2004).

fMRI data were preprocessed using FreeSurfer and FsFast toolkit (V6.0). Functional images were slice‐timing corrected, motion corrected, resampled on the “fsaverage” template of FreeSurfer using the subject T1W image as an intermediate step, smoothed with a 3 mm FWHM Gaussian kernel, intensity normalized, and high‐pass filtered (cut‐off frequency = 1/128 Hz). To ensure that task‐regression steps did not compromise data quality, we computed quality control metrics across all preprocessing pipelines. Temporal signal‐to‐noise ratio (tSNR) was estimated at the ROI level for each pipeline.

### 2.4 Subject‐Level Signal Preparation

Nuisance or unwanted components are usually removed from the fMRI signal before computing the connectivity (Graff et al. 2022; Jiang et al. 2020) via a General Linear Model (GLM) regression. GLM is used to identify the components of the measured temporal signal which are linked to unwanted sources (e.g., motion, non‐cortical tissue, etc.), then the residual fMRI signal is used to compute the functional connectomes.

We filtered the fMRI data with two approaches, one preserving the task‐related information (named “task” condition), and one seeking to remove the task‐related components from the data (named “pseudo‐resting” condition).

- *“Task” condition* (RAW DATA): regressors of the subject‐level GLM of task‐fMRI BOLD time‐series included six motion parameters estimated during realignment, the first five principal components of cerebrospinal fluid (CSF), the first five principal components of white matter (WM) and the global signal mean (Gargouri et al. 2016; Weissenbacher et al. 2009).
- *“Pseudo‐resting” condition* (GLM‐d): the subject level GLM of task‐fMRI BOLD time‐series included the same regressors listed for the “task” condition (i.e., motion, CSF and WM related signals) and the task‐related predictors. Boxcar functions of tasks were convolved with the haemodynamic response function (HRF) implemented in SPM12 toolbox and regressed out from data. Although the preprocessing steps were carried out using a pipeline based on FreeSurfer, task‐related modelling was performed using the haemodynamic response function (HRF) implemented in the SPM12 toolbox. This approach allowed us to model task regressors using a well‐established HRF formulation, including its temporal and dispersion derivatives, which capture potential variability in the shape and timing of the BOLD response. Conventional analysis of fMRI data using GLM employs a neural model with a canonical HRF peaking 5 s after stimulation. The incorporation of further basis functions, namely the canonical HRF temporal and spatial derivatives, accounts for delays and dispersion in the hemodynamic response to neural activity, respectively. For this reason, multiple task‐regression options were included in this analysis: task regression with the canonical HRF (i.e., 0 derivatives), task regression with the canonical HRF plus its temporal derivative (i.e., 1 derivative), and task regression with the canonical HRF plus its temporal and dispersion derivatives (i.e., 2 derivatives), and we named them as GLM‐d0, GLM‐d1, and GLM‐d2, respectively.

### 2.5 Functional Connectivity Estimation

**FIGURE 2:**
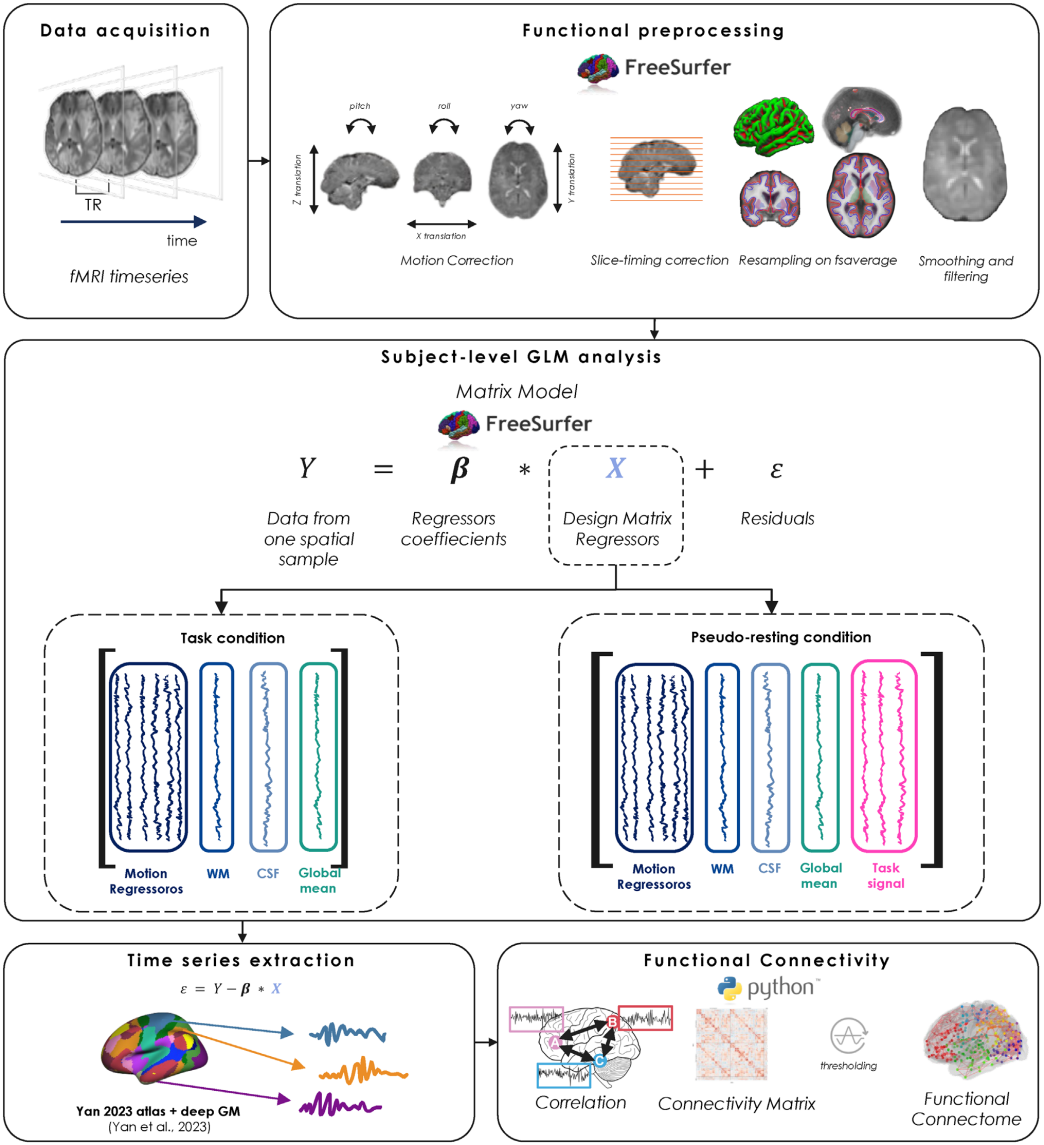
fMRI pipeline. Schematic representation of the pipeline for connectomes. In the preprocessing stream, spatial samples are defined as the vertices of the cortical surface mesh together with the voxels from the 18 regions of deep gray matter. When using the standard FreeSurfer template (fsaverage), this corresponds to 163,842 vertices per hemisphere, totalling 32,684 spatial samples, plus 197,882 voxels from the Freesurfer's “aseg” volumetric segmentation of subcortical structures across the whole brain.

The fMRI time series were resampled following the deep gray matter and cortical gray matter parcellation into ROIs derived from the T1W images. Resampling was performed to improve the signal‐to‐noise ratio and reduce the dimensionality problem (Zalesky et al. 2016). Connectomes were portrayed by means of ROI‐wise Pearson correlation of the entire signal time series of the experiment (i.e., across all conditions of the experiment), resulting in a 218 × 218 symmetric FC matrix for each subject. Connectomes were corrected for population‐based confounders using a linear model at connection level that allowed us to control for the effects of gender, age, IQ, and ADHD‐Inattention removing the amount of the signal explained by these confounders from the dependent variable (i.e., the Pearson correlation of the mean ROI time‐series). The typical pipeline is illustrated in Figure 2.

BOLD signals are influenced by a number of non‐neural processes; therefore, the disposal of spurious connections is a crucial step in neuroimaging connectivity analysis. Thresholding is a popular procedure to sparse the connectivity matrices (He and Evans 2010) even though there is no gold standard algorithm to select the optimal threshold (Ahmadi et al. 2021). Therefore, experiments were conducted with several sparsification thresholds. Specifically, for each thresholding level (th) the top $N\;\left(N=\mathrm{th}*N_{\mathrm{tot}}\right)$ connections were preserved in each subject's connectome, zeroing all the others. Moreover, connections equal to zero in more than half of the subjects were removed also in the other subjects to avoid the issue of multiple removed connections in the group‐level analysis.

### 2.6 Experiment 1: Evaluation of “Pseudo‐Resting” Condition

The first aim of this study is the evaluation of the connectomes derived from the “pseudo‐resting” condition, i.e., obtained by regression of the task related components of the BOLD signal in the fMRI data. In particular, we aim to test whether the “pseudo‐resting” state connectivity is unaffected by the task being performed, as this would allow the integration of datasets derived from different tasks and, potentially, with resting‐state fMRI datasets.

Multiple classification experiments were performed on data preprocessed with the above‐mentioned options (conditions, number of HRF derivatives in the GLM, and different sparsification thresholds) with the aim of predicting the original administered task (i.e., SG or CM) from the derived connectomes. Seven different classifiers were tested with their default settings (i.e., Support Vector Machine (SVM), Logistic Regression, Decision Tree, Random Forest, K‐Nearest Neighbor (K‐NN), Gaussian Naive Bayes, simple Multi‐Layer Perceptron) with a repeated Cross‐Validation (CV) to evaluate the presence of residual task‐related information in the “pseudo‐resting” connectomes. Testing multiple classifiers ensures the independence of the results from the classifier and validates the absence of any correlation between the type of classifier and our data. Cross‐validated accuracy and cross‐validated AUC (Area Under the Curve) were used as performance metrics in this analysis. Thereafter, we employed linear mixed‐effects models to evaluate the effect of preprocessing strategy and sparsity level on the classification metrics. This method allows for both fixed and random effects (e.g., classifier variability). Post hoc comparisons were computed using estimated marginal means with Bonferroni correction for multiple comparisons. A final accuracy close to a random prediction (i.e., 50%) would indicate that the FCs are not different between the two tasks and that a pseudo‐resting state FC can be quantified from task‐fMRI data with an appropriate processing pipeline. Conversely, a larger accuracy would indicate that some task‐related components are still present in the FCs, allowing the classifier to identify the source data. We expect that FCs derived from the “task” condition data will preserve the information related to the task and that the classifiers will successfully discriminate between tasks, while we have no hypothesis about the discrimination on FCs derived from the “pseudo‐resting” condition.

### 2.7 Experiment 2: Impact of the FC Processing Pipeline in a Clinical Context

#### 2.7.1 Classification Experiment and Retrieval of Relevant and Discriminative Connections

The second aim of the present work investigates whether the FC processing pipeline affects subsequent analyses upon behavioral traits, for the purpose of identifying the optimal FC estimate procedure for clinically oriented analyses. We performed a classification experiment with the aim of investigating the impact of FC preprocessing in the characterization of subjects with DD and TRs. On the basis of the results of the previous experiment, we fixed some processing parameters. Specifically, we set the sparsity level to 50% and utilized the HRF without temporal derivatives to remove the task‐related contribution to the signal in the “pseudo‐resting” condition (i.e., the GLM‐d0 approach). Group classification analyses were performed using an SVM classifier (C = 1, linear kernel) with a 6‐folding CV approach to check the validity of the model in describing our data. Significance of classification was tested with a 1000‐permutations test and pairwise differences in the performances of the 4 classification experiments were tested with a two‐tailed homoscedastic *t*‐test. We included a feature selection step to reduce the input dimensionality before training the classifier. In each fold of the cross‐validation procedure, relevant connections to be used as features in the classification were selected by a Mann–Whitney test (*p* < 0.001). Accuracy, AUC and relevant connections were saved to dig in the discriminative process. To assess whether the choice of preprocessing pipeline and the task condition (i.e., SG or CM) influenced classification performances, we used linear mixed‐effects models (LMMs). Separate LMMs were fitted for each performance metric (accuracy and AUC). Connections selected in at least 5 folds out of 6 were identified as stable and discriminative, and compared among the 4 SVM experiments (i.e., task SG, task CM, “pseudo‐resting” SG, and “pseudo‐resting” CM). All connections were grouped according to the 17‐network cortical parcellation described by Thomas Yeo and Sepulcre (2011), plus subcortical regions, which were considered as an additional group, to get an overview of the areas devoted to the discrimination of DD and TR.

#### 2.7.2 On the Interpretation of Discriminative Connections—Forward Models

SVM weights associated with the input connections (i.e., with the features provided to the classification model) identified important features for the classification task, but do not have a direct neurophysiological interpretation: methods for the extraction of neural information from data can be considered as backward models, as they attempt to reverse the data generating process (Haufe et al. 2014). The discriminative weights of linear classifiers, such as support vector machines, reflect the decision boundary and may be influenced by correlations among features, rather than representing the true underlying sources of neural activity. However, as demonstrated by Haufe et al. (2014), linear classification “backward” models can be mathematically transformed into equivalent generative “forward” models. These forward models estimate how the observed data (e.g., functional connectivity patterns) are produced from underlying neural processes, thereby enabling a physiologically meaningful interpretation of the model weights.

To obtain interpretable weights associated with relevant connections, we re‐trained the classifiers of the 4 experiments on the whole dataset and derive the forward model associated with each experiment applying the forward model transformation defined as follows:

$$a=\Sigma_{X}w$$

where w are the weights learned by the classifier (backward model), $\Sigma_{X}$ is the covariance matrix of the input features (i.e., the functional connectivity values of the relevant connections across subjects), and a are the forward model weights associated with each element of the input vector (i.e., the relevant connections). Accordingly, forward connection weights expressed the contribution of the neurodevelopmental condition (i.e., DD) to each element of the feature vector (i.e., relevant connections).

To further enhance interpretability, we grouped connections based on their assigned network group based on the 17‐network parcellation (Thomas Yeo et al. 2011) plus subcortical regions and summed the positive and negative weights. This allowed us to generate summary maps highlighting the brain areas most robustly affected by DD.

We examined the forward‐model weights of functional connections that consistently emerged as relevant across different tasks and preprocessing conditions to highlight robust discriminative features with consistent contributions to group classification.

## 3 Results

Temporal signal‐to‐noise ratio (tSNR) confirmed comparable signal quality across all pipelines, with no evidence of systematic degradation due to task regression (Figure S2).

### 3.1 Experiment 1: Evaluation of Task Derived Resting‐State FC

In the classification experiment aiming to evaluate the presence of task‐related residual components in the FC, all classifiers provided significant classification performances, with accuracy and AUC larger than 50%.

**FIGURE 3:**
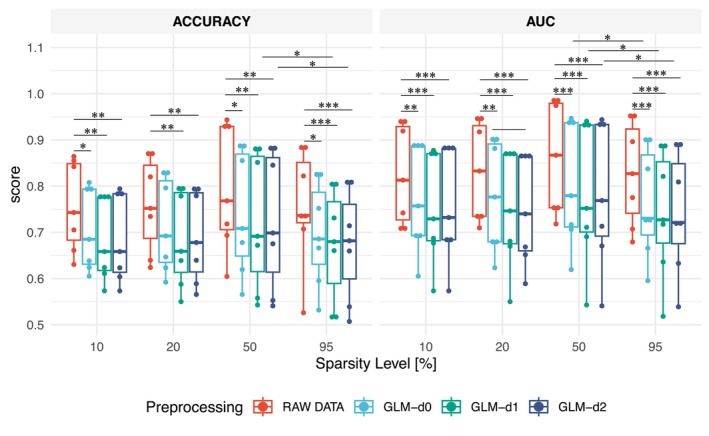
Stimulus inference from connectomes. Task discrimination (SG Vs CM) performances from connectome matrices among the different preprocessing techniques: Applying different GLM derivatives (i.e., no derivatives—RAW DATA, 0 derivative—GLM‐d0, 1 derivative—GLM‐d1, or 2 derivatives—GLM‐d2) and levels of graphs' sparsity (i.e., 10%, 20%, 50%, 95%). Each point of the boxplot represents the average performance of a different classifier. We tested 7 different classifiers, and each one was evaluated via a cross‐validation procedure on a cohort of 154 samples collected from 77 subjects, as each subject performed two distinct tasks, resulting in two samples per subject. Levels of significance (Bonferroni corrected): ^*^ *p* < 0.05, ^**^ *p* < 0.01, ^***^ *p* < 0.001.

As hypothesized, “task” condition (i.e., RAW DATA) provided the best classification performances among the tested preprocessing pipelines, independently of the level of sparsification (Figure 3). Accordingly, task regression did remove task‐related content from the fMRI signal, even though the stimulus can still be inferred from the derived connectomes independently of preprocessing (i.e., sparsification level and number of derivatives included in the HRF). Furthermore, we can advise that a sparsity level of 50% gives the best performances allowing the classifiers to gather the most peculiar information from connectomes in the discrimination of the two tasks, independently of preprocessing (Figure 3).

We fitted two linear mixed models to predict accuracy and AUC using Preprocessing and Sparsity level as fixed effects and the model's classifier as a random effect. The models' total explanatory powers are substantial (conditional R2 are 0.92 and 0.96, respectively). The models' intercepts correspond to Preprocessing = RAW DATA and Sparsity Level = 10%. A significant effect of the *preprocessing* on both classification accuracy and AUC of the tested classifiers was found (Table 2). Moreover, the *sparsity level 50%* shows a significant contribution to both the classification accuracy and AUC (Table 2).

**TABLE 2:** Results of linear mixed effects modeling performed on both accuracy and AUC scores. Each model was fit over a set of 102 samples (7 classifiers × 4 sparsity levels × 4 preprocessing pipelines).

Experiment 1: LMM		Estimate	Std. Error	df	*t*‐value	Pr(>|t|)	
Accuracy	(Intercept)	0.757	0.043	7.184	17.437	< 0.001	***
	PreprocessingGLM‐d0	−0.051	0.019	90.000	−2.712	0.008	**
	PreprocessingGLM‐d1	−0.072	0.019	90.000	−3.836	< 0.001	***
	PreprocessingGLM‐d2	−0.069	0.019	90.000	−3.687	< 0.001	***
	Sparsity_Level20	0.001	0.019	90.000	0.075	0.941	
	Sparsity_Level50	0.041	0.019	90.000	2.184	0.032	*
	Sparsity_Level95	−0.001	0.019	90.000	−0.060	0.953	
AUC	(Intercept)	0.824	0.047	6.541	17.371	< 0.001	***
	PreprocessingGLM‐d0	−0.051	0.014	90.000	−3.588	0.001	***
	PreprocessingGLM‐d1	−0.070	0.014	90.000	−4.878	< 0.001	***
	PreprocessingGLM‐d2	−0.064	0.014	90.000	−4.520	< 0.001	***
	Sparsity_Level20	0.007	0.014	90.000	0.504	0.616	
	Sparsity_Level50	0.038	0.014	90.000	2.662	0.009	**
	Sparsity_Level95	0.003	0.014	90.000	0.175	0.861	

*Note:* Interaction effects are not shown, as they were not significant (all p > 0.3). Level of significance: *p < 0.05, **p < 0.01, ***p < 0.001.

Post hoc pairwise comparisons on fixed effects from the linear mixed‐effects models were performed using estimated marginal means with Bonferroni correction for comparison across preprocessing methods and sparsity levels. Results confirmed significant differences in the classification performances between the “task” (RAW DATA) and the “pseudo‐resting” conditions (GLM‐d0, GLM‐d1, GLM‐d2) in most of the tested sparsification levels both for accuracy and AUC scores, highlighting a clear reduction in discrimination performance when task information is regressed out from fMRI time series.

Specifically, for accuracy, RAW DATA showed significantly higher performance compared to all “pseudo‐resting” regressions at 10% sparsity (*p* < 0.05). Similarly, for AUC, RAW DATA outperformed all “pseudo‐resting” methods across sparsity levels, with *p*‐values consistently below 0.01 or 0.001. No significant differences were found among the pseudo‐resting models (GLM‐d0 vs. GLM‐d1 vs. GLM‐d2) at any sparsity level (all *p* > 0.3), indicating comparable effects among different levels of task regression.

When comparing sparsity levels within each preprocessing method, only a few isolated significant differences emerged. For accuracy, a significant increase was found between 50% and 95% sparsity in GLM‐d1 (*p* = 0.040) and GLM‐d2 (*p* = 0.044). For AUC, significant increases between 50% and 95% were found in GLM‐d0 (*p* = 0.027), GLM‐d1 (*p* = 0.017), and GLM‐d2 (*p* = 0.018), whereas RAW DATA did not show significant differences across sparsity levels (all *p* > 0.05).

Altogether, these results highlight that some task‐related components are still present in the FCs even in the “pseudo‐resting” condition, allowing the classifier to identify the task performed.

### 3.2 Experiment 2: Impact of Connectome Processing on a Clinical Context

**FIGURE 4:**
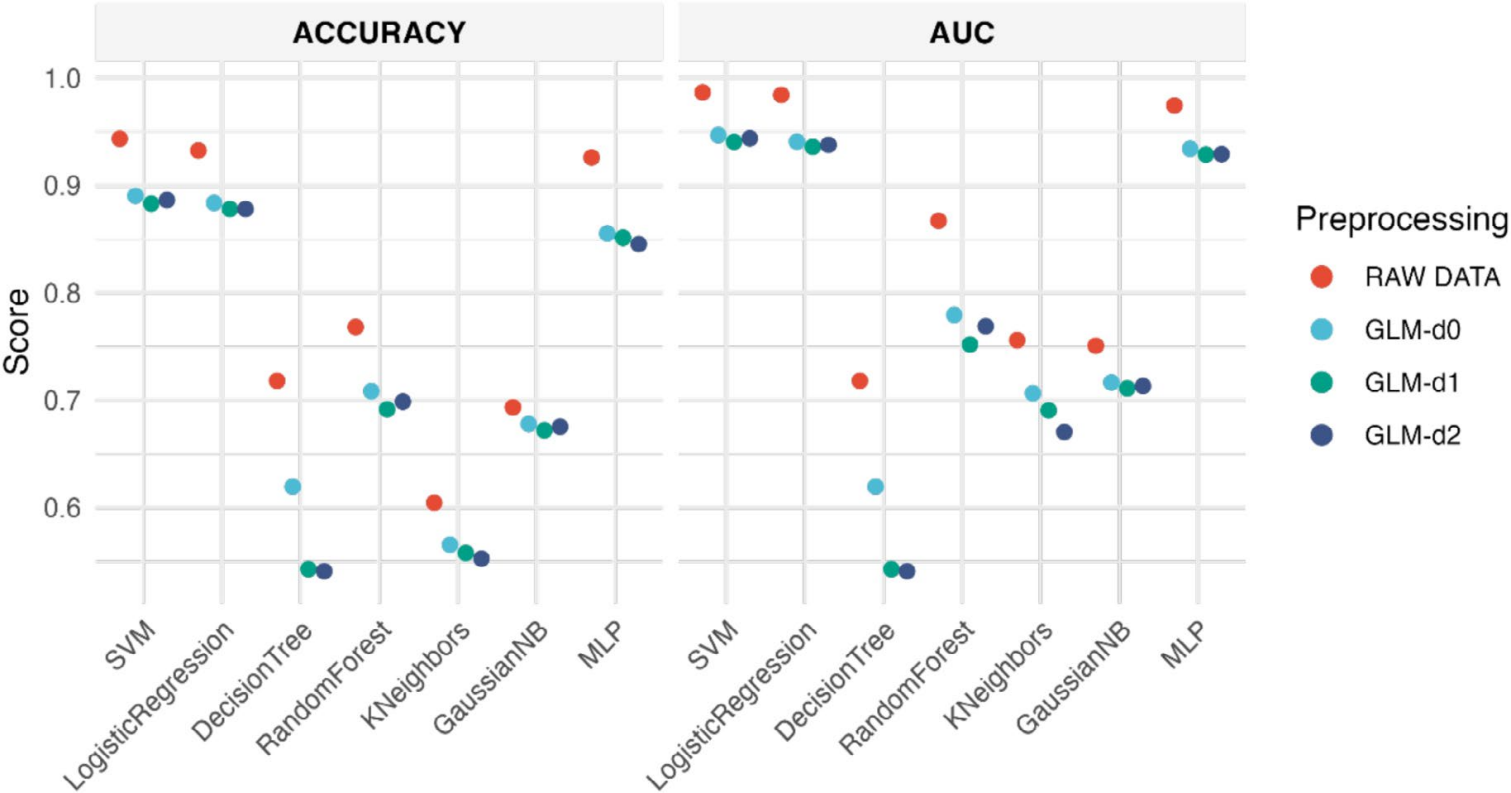
Stimulus inference from connectomes performances at 50% of sparsity level. Cross‐validated accuracy and AUC of classifiers are presented independently from the preprocessing technique.

In *Experiment 2* we set some processing parameters on the basis of the results of *Experiment 1* to reduce the number of different conditions to be considered and simplify the pipeline. More precisely, we found out that (i) a sparsity level of 50% provides the best classification performances in every preprocessing setting; (ii) at 50% sparsification, the SVM classification performs better (Figure 4); and (iii) there is no significant difference in the FCs due to the number of derivatives included in the GLM analysis for the “pseudo‐resting” condition. Therefore, we used only the GLM‐d0 task regression model as “pseudo‐resting” condition to simplify the processing pipeline.

#### 3.2.1 Retrieval of Relevant and Discriminative Connections

The Cross‐Validated metrics of the 4 SVM experiments, and the 1000‐permutations test (*p* < 0.0001) confirmed that classification of connectomes between subjects with DD and TRs is significantly successful in both “task” and “pseudo‐resting” conditions, proving that subject‐specific FCs carry group‐related information (Table 3).

**TABLE 3:** Behavioral trait (DD vs. TR) classification performances from connectome matrices. Experiments were performed using both “task” condition and “pseudo‐resting” condition (GLM‐d0) derived connectomes with a 50% sparsity level. Accuracy and AUC are reported as CV mean ± SD. All performance indices are significantly larger than the random prediction (1000‐permutations test).

	Discriminative connections	ACCURACY MEAN	AUC MEAN
“Task” SG	20	0.78 ± 0.09	0.81 ± 0.09
“Pseudo‐resting” SG	7	0.65 ± 0.10	0.70 ± 0.11
“Task” CM	22	0.73 ± 0.09	0.78 ± 0.13
“Pseudo‐resting” CM	16	0.71 ± 0.14	0.81 ± 0.11

Furthermore, the linear mixed modeling of data showed no significant effect of the processing pipeline or the task (i.e., SG and CM) on the classification performances (Table 4).

**TABLE 4:** Linear mixed model results testing for the influence of condition and task (i.e., SG or CM) upon classification performances.

Experiment 2: LMM		Estimate	Std. Error	df	*t* value	Pr(>|t|)	
Accuracy	(Intercept)	0.801	0.042	15.70	19.054	< 0.001	***
	preprocessing “task”	0.026	0.043	16	0.601	0.556	
	taskSG	−0.002	0.043	16	−0.050	0.961	
AUC	(Intercept)	0.876	0.041	12.33	21.276	< 0.001	***
	preprocessing “task”	0.005	0.037	16	0.143	0.888	
	taskSG	0.033	0.037	16	0.893	0.385	

*Note:* Level of significance. *** *p* < 0.001.

**FIGURE 5:**
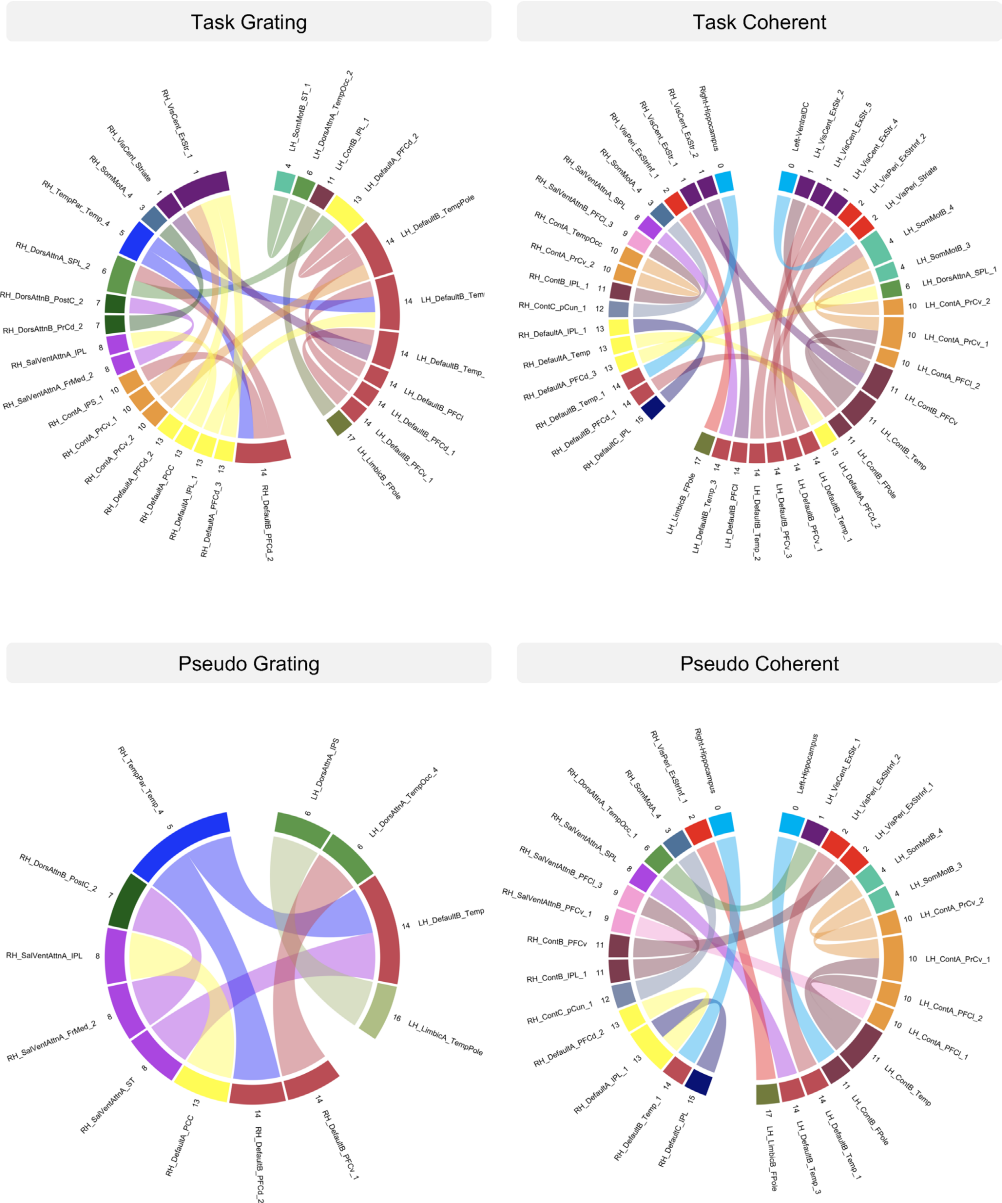
Chord Diagrams of relevant connections in the 4 classification experiments. Each graph portrays the relevant connections selected as features by the classifier in the SVC experiments. Connections were intended as relevant if they were selected by the CV‐classifier at least 80% of the times (i.e., in 5 folds over 6). ROIs color‐coded according to their corresponding macro‐region (matched to the assigned 17‐network parcellation or subcortical regions). Specifically, each macro‐region (e.g., visual, frontal, parietal) was assigned a distinct color, consistently with the scheme used in Figure 1. In total, 20 connections were selected in the “task” SG experiment, 7 in the “pseudo‐resting” SG experiment, 22 in the “task” CM experiment, and 16 in the “pseudo‐resting” CM experiment. Additionally, the dGM network is represented using the number “0” and is colored in cyan.

To better illustrate the relationship between the ROIs linked by the classifier‐selected FC connections in the two preprocessing conditions (i.e., “task” and “pseudo‐resting” conditions), we used circular plots to display the preprocessing‐modulated connections (Figure 5).

A color code was used to identify the networks involved by the small ROIs, i.e., ROIs belonging to the same network are depicted by the same color. Figure 5 provides a visual representation of the connections selected by the classifier. It can be noted how the number of discriminative connections lowers when the task information is regressed out of the signal, highlighting the networks able to discriminate between subjects with DD and TRs above and beyond the connections promoted by the task‐specific stimuli. Thus, the “pseudo‐resting” condition reveals functional connections linked to the clinical disorder and not necessarily to the task. More in detail, to explore the impact of preprocessing (“task” Vs. “pseudo‐resting” conditions) and the type of task (SG vs. CM) in the discriminative process of the classifiers, we quantified the overlap in the relevant connections which were shared among the different classification experiments. No connections overlapped between the classifiers working on SG and CM, neither in “task” or “pseudo‐resting” (*intra‐processing* and *inter‐task*). On the contrary, a large agreement is achieved when comparing “task” and “pseudo‐resting” conditions in both SG (4 connections, 57% of the maximum number of shareable connections) and CM (11 connections, 68% of the maximum number of sharable connections) (*intra‐task* and *inter‐processing*).

We grouped the relevant connections on the basis of the Yeo 2011 networks and on the basis of their intra/inter hemispheric nature, thus highlighting the possible high‐level impact of DD on functional connectivity. Table 5 shows the number of relevant connections targeting each network in each of the four experiments.

**TABLE 5:** Analysis of the macro‐regions linked by the connections involved in the classification. For each classification experiment we report the number of the selected connections linking the given macro‐regions. We have color‐coded the cells in the table using a graded color scale, where increasing intensity corresponds to a higher number of connections.

Network	“Task” SG	“Task” CM	“Pseudo‐resting” SG	“Pseudo‐resting” CM	Sinusoidal gratings [average]	Coherent motion [average]
*Subcortical structures*	0	2	0	2	0	2
*Visual A*	4	5	0	1	2	3
*Visual B*	0	3	0	3	0	3
*Somatomotor A*	1	1	0	1	0.5	1
*Somatomotor B*	1	3	0	2	0.5	2.5
*Temporal Parietal*	2	0	2	0	2	0
*Dorsal Attention A*	3	1	2	1	2.5	1
*Dorsal Attention B*	2	0	1	0	1.5	0
*Salience/VenAttn A*	2	1	3	1	2.5	1
*Salience/VenAttn B*	0	1	0	2	0	1.5
*Control A*	3	6	0	5	1.5	5.5
*Control B*	1	6	0	5	0.5	5.5
*Control C*	0	1	0	1	0	1
*Default A*	6	4	1	3	3.5	3.5
*Default B*	14	8	4	3	9	5.5
*Default C*	0	1	0	1	0	1
*Limbic A*	0	0	1	0	0.5	0
*Limbic B*	1	1	0	1	0.5	1

For a surface‐based representation of this analysis see Figure S1. Generally speaking, we found a strong *inter‐processing* correlation in the relevant distribution of connections within each task (i.e., *ρ* = 0.70 (*p* = 0.0012) for the SG and *ρ* = 0.80 (*p* < 0.001) for the CM) and a moderate *inter‐task* correlation within each preprocessing pipeline (i.e., *ρ* = 0.67, *p* < 0.05) for the “task” condition and a negative, although non‐significant, correlation (*ρ* = −0.16, *p* = 0.53 for the “pseudo‐resting” condition). Because of the strong *intra‐task* correlation, we averaged the number of connections linking the networks for each task across the preprocessing techniques to highlight the connected networks on which DD could have an effect. The most frequently targeted networks for both the SG and the CM are part of the “Default B” and “Default A” networks, which link the Inferior Frontal Cortex, the Inferior Parietal Cortex, the Dorsolateral Prefrontal Cortex and the Orbital and Polar Frontal Cortex. Similarly, the CM targets the “Control A” and “Control B” networks, which mainly link the Inferior Parietal Cortex, the Dorsolateral Prefrontal Cortex and the Orbital and Polar Frontal Cortex. All these brain areas are known to be involved in DD.

#### 3.2.2 On the Interpretation of Discriminative Connections—Forward Models

**FIGURE 6:**
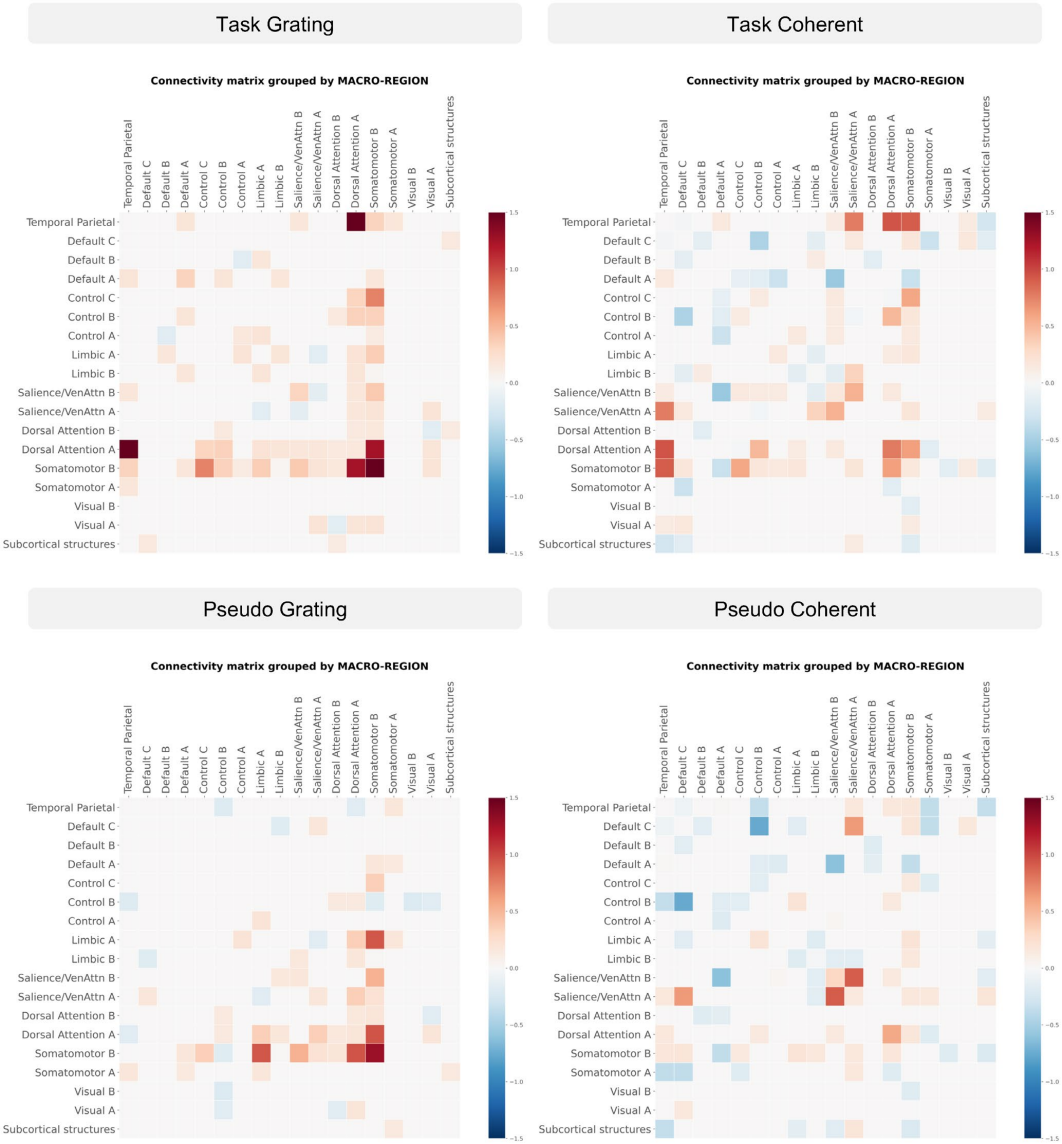
Macro‐regions connectomes. Macro‐regions connectivity matrices of forward weights for SG and CM. For each condition, we grouped the relevant functional connections based on their associated macro‐regions and computed the sum of their positive and negative forward‐model weights.

We wanted to inspect how the preprocessing of the fMRI data would affect the clinical classification by looking at the forward weights associated with connections derived by the forward models. For each condition, we grouped the relevant functional connections based on their associated macro‐regions and computed the sum of their positive and negative forward‐model weights. This allowed us to visualize which brain areas were most strongly associated with group differences, providing an interpretable overview of the regions most affected in developmental dyslexia. Figure 6 shows the macro‐regions connectivity matrices illustrating the contribution of DD in each macro‐region that we used to visually compare the preprocessing of this analysis, and Table 6 reports the pairwise correlations and relative *p*‐values of the generative weights (i.e., from the forward model) associated with the relevant connections shared between conditions. We do not report or further interpret correlation values obtained from comparisons with *N_common* < 10, as these estimates are based on too few shared elements and thus lack statistical and interpretative validity. As a result, we can state that even when we move to the clinical context, preprocessing does not affect the clinical results.

**TABLE 6:** Correlations and associated *p*‐values of weights derived by the forward models for overlapping connections between conditions. To obtain interpretable weights for relevant the model was re‐trained on the entire dataset.

Condition A	Condition B	N_common	Correlation	*p*
*“Task” SG*	*“Pseudo‐resting” SG*	39	0.98	< 0.001
*“Task” CM*	*“Pseudo‐resting” CM*	44	1.00	< 0.001

## 4 Discussion

In this work, we investigated the impact of applying a processing pipeline typical of rs‐fMRI to task‐based fMRI data. Our study had two main objectives: first, to determine whether task‐based fMRI data could be used to derive connectomes independent from the original task executed and therefore comparable to the resting‐state connectomes; second, to explore how different processing pipelines affect the final connectivity measures describing patterns linked to some clinical conditions.

Our first experiment showed that different classifiers could discriminate between two distinct tasks administered to the same subject cohort on the basis of the derived connectivity matrices, regardless of the processing pipeline used. More in detail, regression with task predictors reduced the ability to distinguish between two visual tasks (i.e., SG and CM), indicating that GLM regression effectively removed part of the task information from the fMRI signal. However, classifiers were still able to successfully discriminate between the two tasks, even after task regression. This suggest that connectomes retain some task‐related signal even after different task regression approaches. This finding echo previous work by Cole et al. (2014), who also observed that task regression only modestly affected FC estimates, and by Elliott et al. (2019), who noted that pipeline decisions, including whether or not to regress out task signals, had meaningful but limited influence on predictions of behavioral or cognitive traits. Our findings contribute to this ongoing discussion by demonstrating that post‐regression connectomes still contain task‐dependent structure, likely reflecting not just task‐evoked activations but also broader task‐induced states. This supports the idea that functional networks are continuously interacting with each other at rest, with the same functional hierarchy that is seen during action and cognition (as argued in Tavor et al. 2016) and suggests that connectomes retain meaningful task‐related variance even after attempts to isolate spontaneous neural fluctuations. While residual task‐specific information may reflect persistent brain states beyond what standard GLM regressors capture, we also considered the possibility of regression‐related artifacts. To address this, we examined the variance and distribution of residual signals and found no evidence of distortions introduced by task regression (see Figure S3). These results suggest that the observed classifier performance is unlikely to stem from spurious artifacts, but rather from meaningful differences retained after regression. What remains unexplored post‐regression is the extent to which variations in connectivity are directly driven by task execution (i.e., due to cortical activations induced by the task) or indirectly linked to the task (i.e., related to a shift in synchronization between brain areas as a result of the task recruiting one of them). Additionally, the number of derivatives in the GLM had a minor and non‐significant effect on the outcomes, suggesting that adding derivatives does not improve the removal of task‐related signal from the connectivity measures (i.e., in measures of temporal synchronization between brain areas). Taken together, these results confirm that connectivity measures derived from task fMRI data (i) are definitely dependent on the original task and, therefore, (ii) they are not comparable to those derived from the resting‐state fMRI data. In our second experiment we compared the ability of the FCs derived with different processing pipelines to identify group‐specific connectivity patterns. Our findings showed no significant differences in group classification performances between FC assessed with or without task regression. This suggests that temporal signal components directly induced by the task do not affect the discrimination between children with DD and TRs, and that signal preprocessing does not influence the way the classification rules are inferred in the training, leading to similar evaluation of relevant features. By examining the connected brain areas involved in the classification, we found that more than half of the discriminative and relevant connections were shared across different processing pipelines for each task (i.e., SG and CM). The type of task influenced how the classifier discriminates between subjects with DD and TRs, as relevant connections depend on the task performed in both “task” and “pseudo‐resting” conditions (i.e., SG and CM share no connections). This indicates that “pseudo‐resting” data also exhibit attributes linked to the type of task, and that the functional architecture evoked during the task dominates over preprocessing decisions. To compare the outcome of the group classifications across the processing pipelines in relation to the clinical disorder (in our example, DD), we used forward models derived from the trained classifiers, confirming that processing had no impact on the interpretation of clinical results. Furthermore, correlation analysis showed stronger relationships within tasks than between different processing pipelines, even when task‐related signals were regressed out. These findings suggest that rather than reflecting synchronization between regions specifically activated by the task, the connectivity derived from task‐based fMRI data is more associated with a general state induced by the task, likely driven by the temporal structure shared across the task. Additionally, this implies that task‐derived connectivity analysis could reveal distinct aspects of task‐based fMRI data independent of direct brain activations.

However, substantial confusion and debate persist regarding connectivity‐oriented processing of task‐based fMRI data. In some cases, this has led researchers to perform two versions of their analysis, one with task regression and one without (Elliott et al. 2019; Cole et al. 2014). In their work, Cole et al. (2014) used task regression to remove task‐activation variance and reduce the likelihood of functional connectivity estimates being influenced by simple co‐activation. They presented only the results obtained after task‐regression, observing minimal effects of this preprocessing step on their findings, similar to our results. Similarly, Elliott et al. (2019) adopted a data‐driven approach to pipeline selection and prioritized results obtained with task regression, noting a greater alignment between their results and the clinical outcomes when predicting cognitive ability. The ongoing debate surrounding task‐based connectivity processing in fMRI highlights the need for continued exploration and consensus‐building within the scientific community to establish best practices in this area.

Many potential analyses related to this topic were not investigated in this work, leaving opportunities for future work. For instance, future analyses should include datasets with both task‐based and resting‐state data for a direct comparison of the connectomes. Furthermore, we used a retrospective dataset including tasks with different durations (11′30″ for SG and 9′30″ for CM) and design (SG is a block‐design task, CM is an event‐related task), always administered in the same order (SG was acquired first and a structural sequence was acquired between the two fMRI sequences). We cannot exclude the possibility that any of these aspects had an impact on the classification experiments, even if it sounds unlikely. Nevertheless, our investigation confirms that a resting‐state‐like FC cannot be derived from task‐based data and that task‐FC, whether with or without task signal regression, remains linked to the task. It is therefore reasonable to conclude that it is not possible to derive a true resting‐state FC from task‐based fMRI data.

Finally, our focus was limited to Pearson correlation‐based static FC. Future work could examine whether alternative connectivity metrics (e.g., psychophysiological interactions, dynamic FC, or directed connectivity models) exhibit similar resilience or sensitivity to preprocessing strategies.

## 5 Conclusions

In this study, we analyzed the preprocessing of task fMRI data to explore the contribution of task information to connectomics. The successful discrimination of the performed tasks using the “pseudo‐resting” pipeline indicates that connectomes preserve task‐related information even after GLM regression, suggesting that a task‐free “pseudo‐resting” state cannot be inferred from task fMRI data.

However, we also demonstrated that connectomes derived from task‐fMRI data can still be used to investigate clinical conditions and their impact on brain connectivity, likely independent of task regression.

Moreover, task‐regressed connectomes, which reduce the number of relevant connections, may provide insights into behavioral traits not explicitly linked to the task‐driven stimuli.

## Author Contributions

**Alice Giubergia:** conceptualization, formal analysis, methodology, writing – original draft, writing – review and editing. **Sara Mascheretti:** supervision, writing – review and editing. **Valentina Lampis:** writing – review and editing. **Tommaso Ciceri:** writing – review and editing. **Martina Villa:** resources. **Chiara Andreola:** resources. **Filippo Arrigoni:** resources. **Alessandra Bertoldo:** supervision, writing – review and editing. **Denis Peruzzo:** supervision, conceptualization, writing – review and editing, funding acquisition.

## Funding

This work was supported by the Italian Ministry of Health.

## Ethics Statement

The study was reviewed and approved by the Ethics Committee of the Scientific Institute, IRCCS Eugenio Medea.

## Consent

Informed consent was obtained from all subjects involved in the study.

## Conflicts of Interest

The authors declare no conflicts of interest.

## Supporting information


**Figure S1:** Surface‐based representation of the macro‐ROI linked by the connections involved in the classification. For each classification experiment we show the number of the selected connections linking the given macro‐ROI.
**Figure S2:** Temporal signal‐to‐noise ratio (tSNR) distributions computed at the ROI level for each preprocessing pipeline. Comparable tSNR values were observed across pipelines, indicating that task‐regression procedures did not degrade signal quality. tSNR was computed as the ratio between the mean and the standard deviation of the time activity curve, for each sampled ROI.
**Figure S3:** Time course activity variance. The chart shows a boxplot of the ROI‐wise mean variance of the time courses of the two fMRI tasks (i.e., Sinusoidal Grating and Coherent Motion) across the different preprocessing pipelines.

## Data Availability

The extracted ROI‐wise time series supporting the findings of this study are available on request from the corresponding author. The data are not publicly available due to privacy or ethical restrictions.
