# Supplementary material for: An Examination of Task‐Evoked fMRI Data Processing in Functional Connectivity
Source: J Neurosci Res. 2026 Jun 9;104(6):e70132. doi: 10.1002/jnr.70132 (PMC13250240; doi:10.1002/jnr.70132)
Supplement: Supplementary file 1 — Figure S1: Surface‐based representation of the macro‐ROI linked by the connections involved in the classification. For each classification experiment we show the number of the selected connections linking the given macro‐ROI. Figure S2: Temporal signal‐to‐noise ratio (tSNR) distributions computed at the ROI level for each preprocessing pipeline. Comparable tSNR values were observed across pipelines, indicating that task‐regression procedures did not degrade signal quality. tSNR was computed as the ratio between the mean and the standard deviation of the time activity curve, for each sampled ROI. Figure S3: Time course activity variance. The chart shows a boxplot of the ROI‐wise mean variance of the time courses of the two fMRI tasks (i.e., Sinusoidal Grating and Coherent Motion) across the different preprocessing pipelines. [file JNR-104-e70132-s001.docx]

Supplementary Material


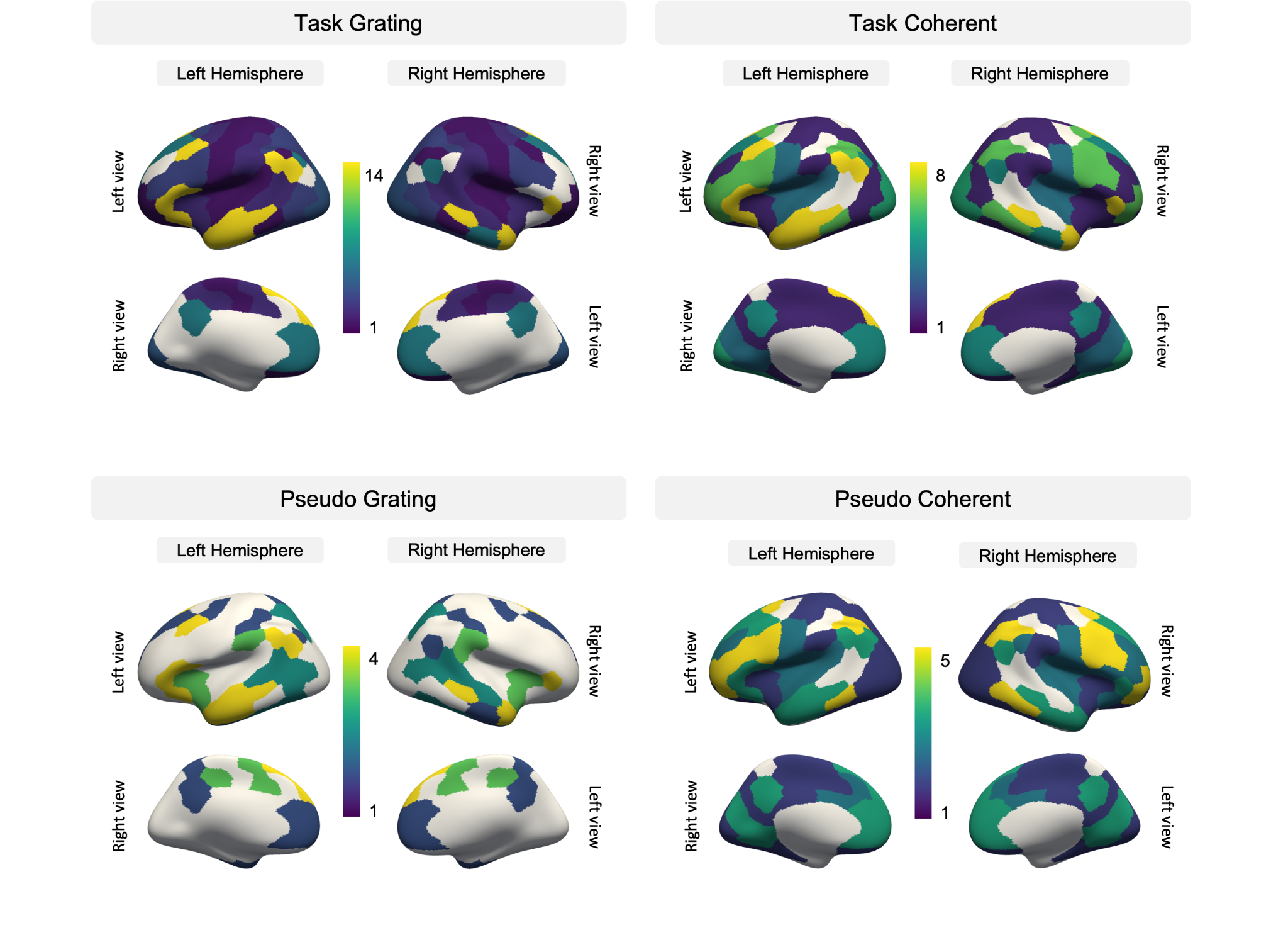
**Figure S1**. Surface-based representation of the macro-ROI linked by the connections involved in the classification. For each classification experiment we show the number of the selected connections linking the given macro-ROI.


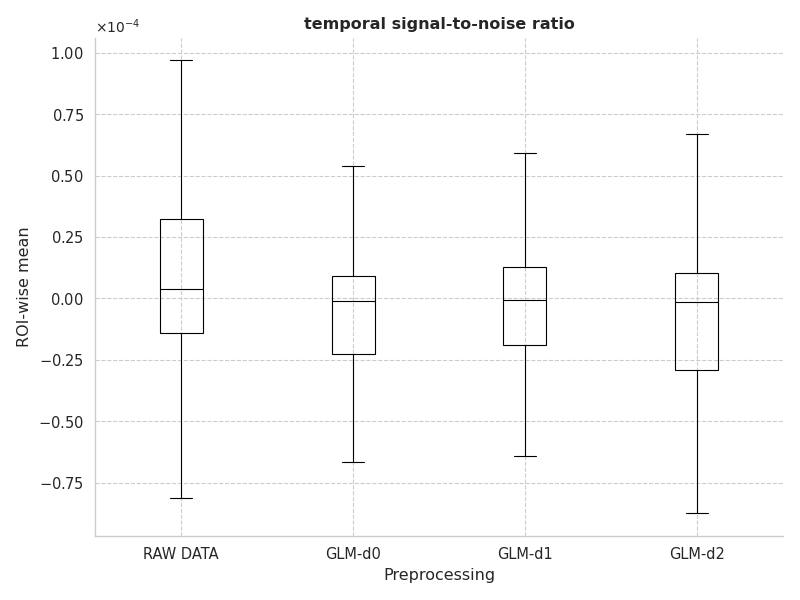
**Figure S2.** Temporal signal-to-noise ratio (tSNR) distributions computed at the ROI level for each preprocessing pipeline. Comparable tSNR values were observed across pipelines, indicating that task-regression procedures did not degrade signal quality. tSNR was computed as the ratio between the mean and the standard deviation of the time activity curve, for each sampled ROI.

**Figure S3. Time course activity Variance**. The chart shows a boxplot of the ROI-wise mean variance of the time courses of the two fMRI tasks (i.e., Sinusoidal Grating and Coherent Motion) across the different preprocessing pipelines


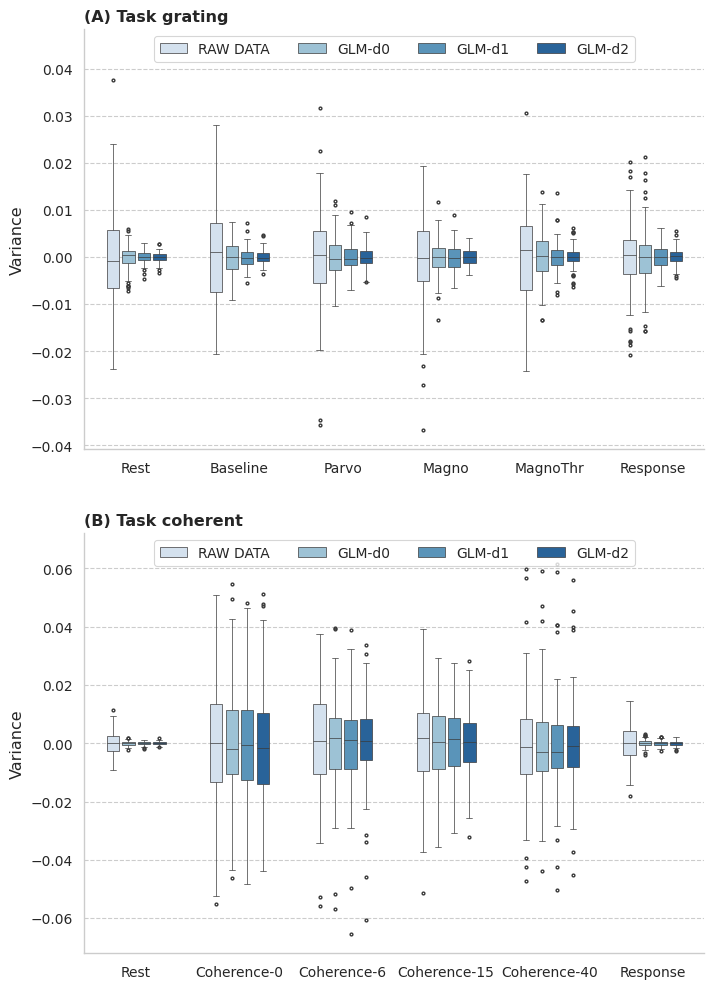


**Complementary Bayesian Statistical Analysis**

We include Bayesian statistics alongside traditional inferential analyses, in order to better quantify the likelihood of absence of effects of our experiments. We performed Bayesian analyses using the Region of Practical Equivalence (ROPE) approach.

Table “Bayesian statistics of Experiment 1” and Table “Bayesian statistics of experiment 2” report the results of the Bayesian model, including the median posterior estimates, 95% credible intervals (CI), the probability of direction (PD), and the proportion of the posterior distribution falling within the region of practical equivalence (ROPE).

**Table Bayesian statistics of Experiment 1**. Bayesian statistics for the effect of the preprocessing pipeline.

**Table Bayesian statistics of Experiment 2.** Bayesian statistics for the effect of the clinical context.

In particular, our Bayesian results on Experiment 1, complementing Table 2 in the Manuscript, indicate that the preprocessing effects (GLM-d0, GLM-d1, GLM-d2) showed negative medians with narrow credible intervals excluding zero, pd = 100%, and 0% in ROPE. These results provide strong evidence that each GLM preprocessing pipeline significantly reduced performance relative to the reference level, in both Accuracy and AUC.

The effect of Sparsity level 20% had a median close to zero, wide CIs, low pd (64.88% for Accuracy; 55.74% for AUC), and a large proportion within ROPE (73.03% and 89.74%). This suggests high uncertainty and strong evidence for a practically null effect. In contrast, Sparsity level 50% showed positive effects (Accuracy: 0.04, AUC: 0.03) with narrow CIs, pd = 100%, and 0% in ROPE—indicating a reliably beneficial effect on classification performance. The effect of Sparsity level 95% was negative and small in magnitude, with moderate-to-high pd (84–91%) but more than 50% of the posterior within ROPE, suggesting only weak evidence for a negative effect, and high probability of practical irrelevance.

The Bayesian results on Experiment 2 complementing Table 4 indicate that for the effect of condition, the posterior distributions had medians close to zero (Accuracy: 0.03, AUC: 0.0049), wide credible intervals including zero, moderate pd (69.9% and 54.3%, respectively), and substantial portions of the distribution within ROPE (14–18%). These results suggest weak or negligible evidence for a condition effect. Similarly, for the task “SG” effect, both Accuracy and AUC showed high uncertainty (CIs spanning zero), low-to-moderate pd (51–76%), and notable percentages within ROPE (>13%), again indicating limited evidence for a meaningful influence of task.

These Bayesian analyses complement the frequentist results by offering a more nuanced interpretation of null findings, reinforcing that the observed non-significant effects are likely true null effects rather than inconclusive results due to limited power.
